# A Longitudinal Analysis of Black Box Warnings: Trends and Implications for Drug Safety

**DOI:** 10.7759/cureus.57597

**Published:** 2024-04-04

**Authors:** Yazhini Rajendran, Nikhila Kondampati, Madhavi Eerike, Kalpana Mali, Leo Francis C

**Affiliations:** 1 Pharmacology, All India Institute of Medical Sciences, Bibinagar, Hyderabad, IND

**Keywords:** post-marketing studies, adverse events (ae), black box warnings, drug label, drugs

## Abstract

A black box warning, signaling potential life-threatening adverse effects of medications or medical devices, is crucial for public and healthcare professional awareness. Comprehending and adhering to these warnings can prevent serious harm. This review aims to elucidate their significance. Data on drugs with black box warnings were collected from the Food and Drug Administration's (FDA's) official website using the search term 'Boxed warnings' from January 1, 2015, to January 31, 2024. A Microsoft Excel spreadsheet (Microsoft Corporation, Redmond, WA, USA) containing black box warnings for this period was downloaded from the FDA's website. Additional parameters, such as drug class and whether the warnings were new or existing, were added to the downloaded spreadsheet. The collected data were organized by year, categorizing new and existing warnings, along with details on the evidence source, system-wise classification, and black box warnings for commonly used drugs, including their clinical significance. Results show that in the past decade, 40% of black box warnings were issued in 2023, followed by 12% in 2022. Most warnings (67%) comprised existing ones with minor revisions while 29% were new. Nine existing warnings were removed during the period. Post-marketing studies predominantly provided evidence for these warnings. Neuropsychiatric concerns like addiction potential (31%), suicidal tendency (7%), and hypersensitivity reactions (12%) were the frequently encountered black box warnings. Black box warnings play a crucial role in highlighting the serious adverse effects of medications. Neuropsychiatric warnings have been frequent over the past decade. Awareness of these warnings is essential to prevent adverse effects and enhance patient care, especially concerning drugs like guaifenesin/hydrocodone bitartrate, zolpidem, and montelukast commonly encountered in clinical practice.

## Introduction and background

A boxed warning, sometimes known as a black box warning, alerts medical professionals and the general public to the possible significant and life-threatening adverse effects of medications that could result in harm or death. The Food and Drug Administration (FDA) initially introduced it in 1979 [1]. The name "boxed warning" originates from the prominent black border surrounding FDA-specified cautionary information on drug labels. The content in the box needs to be organized in a bulleted fashion with a header written in bold uppercase letters [2]. The medicine label, also known as the package insert or product information, is the written, printed, or visual material on the drug product's container. It includes an overview of the necessary scientific data from studies and observations made after a medication is put on the market to support its safe and efficient use [3]. Code of Federal Regulations (CFR) "Warnings (21CFR 201.57 (e)")" states that "Labeling shall explain major adverse reactions and potential safety hazards, limits in use imposed by them, and steps that should be taken if they occur." This is how the black box warning is defined. As soon as there is plausible information linking a drug to a significant risk, the labeling must be updated to add a warning; a causal connection need not be established. Additionally, it is mentioned that while human data are typically the basis for boxed warnings, proof of significant animal toxicity studies may be used in its place [4]. When there is established clinical evidence showing a drug's hazards are less than those of prior trials, the FDA may decide to withdraw the black box warnings that have been issued [1]. But as of now, there are no appropriate rules for eliminating black box alerts [5].

Black box warning process

**Figure 1:**
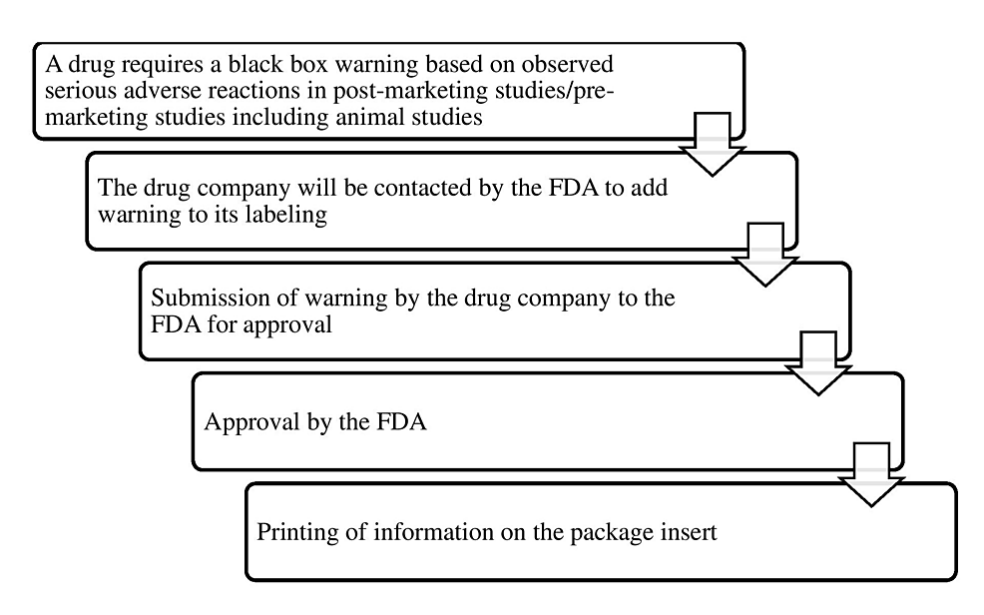
Process of black box warning Source: [1] The figure is not republished work. The content is extracted from the cited reference and condensed in the form of a figure by the authors.

The process of assigning a black box warning to a drug to printing it on the package insert is summarized as a flowchart in Figure 1.

A serious adverse event can be avoided by the appropriate use of the drug, like avoiding it in certain populations or specific situations with the help of a black box warning. By avoiding the serious adverse effects, the cost of health care can be reduced and the quality of life of patients can be maintained. It aids in warning the public and alerting healthcare providers to the cautious use of a particular medical product [1]. Monitoring strategies can be streamlined for drugs with black box warnings. However, the downside of this warning includes a lack of compliance by the doctors and less transparency on the process of black box warnings. Drugs that were approved by fast-track status are more likely to receive a boxed warning after they hit the market because of less time for safety analyses [6]. The common reason for less compliance by doctors is that the warnings issued by the FDA are numerous and the information gets pooled up without reaching the treating physicians.

There are more than 1000 articles available on black box warnings for a particular class of drugs. Yet, there is a lack of consolidated reports of all the black box warnings for drugs issued in the last 10 years. Considering the COVID-19 pandemic during this period and its resulting inadvertent use of many drugs, this study period was chosen. Thus, this systematic review aims to fulfill the aforementioned lacunae.

## Review

Materials and methods

Information related to drugs with black box warnings was retrieved from the official website of the FDA (https://www.accessdata.fda.gov/scripts/cder/safetylabelingchanges/index.cfm) [7] under the ‘Drugs safety related Labeling Changes’ (SrLC) of ‘Drug Approvals and Databases’ using the search term ‘Boxed warnings’ section for the period January 1, 2015, to January 31, 2024, intending to provide a consolidated report of the black box warnings. This review aims to retrieve and segregate the black box warnings issued in the last decade with their clinical significance. Table 1 lists the inclusion and exclusion criteria for the review.

**Table 1:** Inclusion and exclusion criteria

S.No	Inclusion criteria
1.	Boxed warnings released on the official FDA website from January 1, 2015, to January 31, 2024
2.	Drugs reported with black box warnings, either existing or revised
S.No	Exclusion criteria
1.	Warnings and precautions
2.	Contraindications
3.	Drug Interactions
4.	Patient counseling information/patient information/patient guide
5.	Adverse reactions
6.	Use in a specific population
7.	Boxed warnings with incomplete information
8.	Repetition of the same boxed warnings for the same drug
9.	Black box warnings issued before 2015 and reintroduced with minor revisions

Data collection and statistical analysis

A Microsoft Excel spreadsheet (Microsoft Corporation, Redmond, WA, USA) containing black box warnings for the study period was downloaded from the US FDA official website [7]. Additional parameters like the class of the drugs and new and existing black box warnings were added to the Excel sheet. The data were summarized using descriptive statistics expressed in percentages (%) or proportions as period-wise categorization of black box warnings, new and existing/revised, removed, drug class, types, and clinical significance.

Results

**Figure 2:**
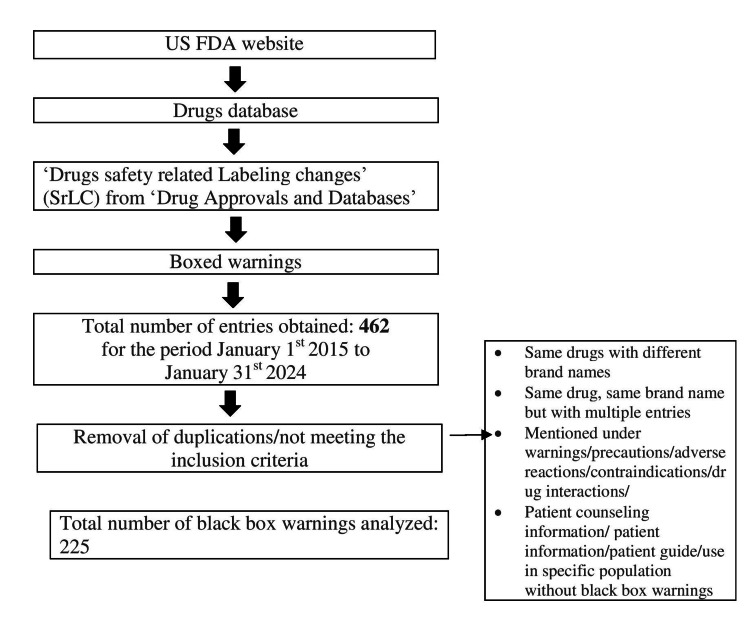
The data extraction workflow Image created by the authors

Figure 2 depicts the data retrieval process undertaken for this review. A total of 462 entries of black box warnings were available in the downloaded material for a decade from the US FDA official website [7]. Of these, 225 were included in this review after removing entries as per the exclusion criteria. The collected data were presented as black box warnings issued each year, starting from 2015 to 2024, new and existing black box warnings, black box warnings according to the source of evidence, system-wise classification of black box warnings, and black box warnings issued for commonly used drugs with their clinical significance.

Period-wise classification of black box warnings

**Figure 3:**
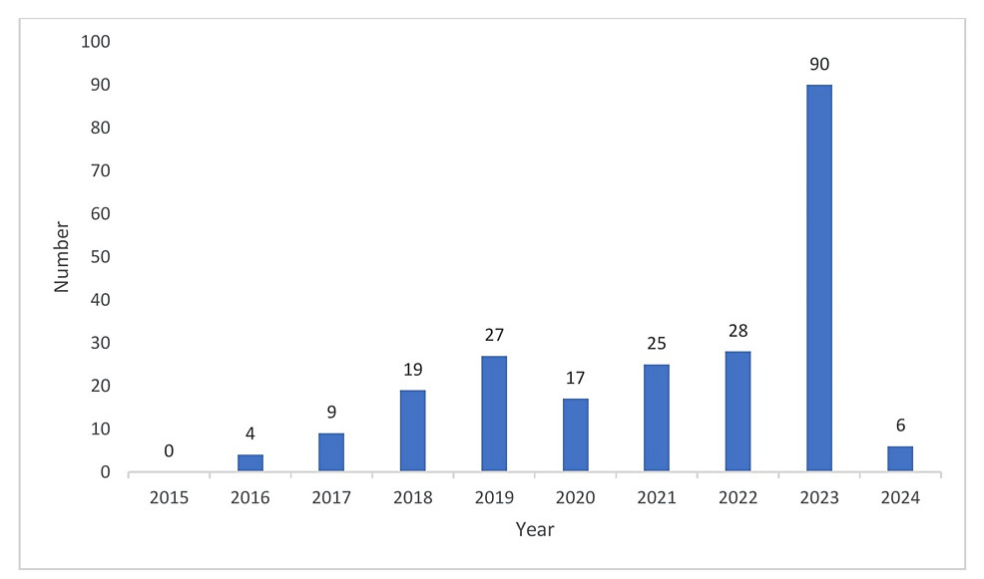
Year-wise distribution of black box warnings Source: [7] The figure is not republished work. The content is extracted from the cited reference and condensed in the form of a figure by the authors.

Figure 3 puts forth the year-wise distribution of black box warnings. There was a maximum number of warnings issued in the year 2023 (40%) followed by 2022 (12%) [7]. Though the exact reason could not be made out, the ascending trend in the number of black box warnings, potentially reveals the increased scrutiny and drug safety monitoring.

Category of black box warnings with their source of evidence

Table 2 presents the categorization of the issued black box warnings in the last decade as newly issued warnings, existing warnings with minor revisions, and removal of the existing warnings and their source of evidence as pre-marketing studies, animal studies specifically, and post-marketing studies [7].

**Table 2:** Category of black box warnings and their source of evidence Source: [7] The extracted data from the cited source was summarized in the table by the authors.

S. No	Black box warnings	n (%)
1.	New	65 (29%)
2.	Existing and revised	151 (67%)
3.	Removed	9 (4%)
S.No	Based on the source of evidence	n (%)
1.	Post-marketing studies	169 (78%)
2.	Pre-marketing studies	42 (19%)
3.	Animal studies	5 (2%)

Classification of drugs with black box warnings

**Figure 4:**
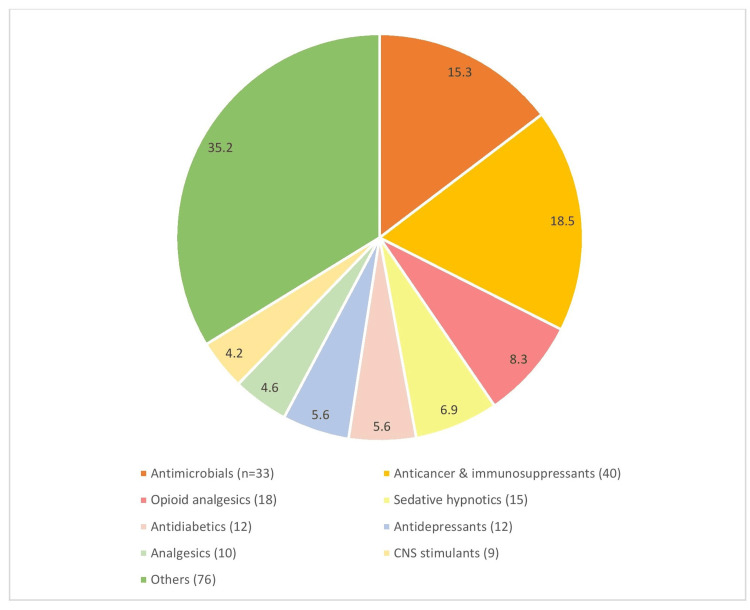
Categorization of drugs as per the class [7] The figure is not republished work. The content is extracted from the cited reference and condensed in the form of a figure by the authors.

Figure 4 presents the black box warnings that were classified according to the class of drugs with percentages in the chart and the number of drugs being mentioned in the legends. ‘Others’ include miscellaneous drugs such as recombinant enzymes, hormone analogs, radiocontrast diagnostic agents, etc. [7].

Types of black box warnings

Table 3 depicts the extracted black box warnings structured for better perception [7]. Drug addiction contributed to the majority of the black box warnings and those with single entries, such as dehydration, risk of radiation exposure, drug interactions, etc., were the least number of black box warnings.

**Table 3:** Types of black box warnings Source: [7] The results were obtained from the cited source.

S.No	Black box warnings	n (%)
1.	Birth defects	15 (7%)
2.	Cardiovascular events	14 (6%)
3.	Hypersensitivity reactions	26 (12%)
4.	Infections	21 (10%)
5.	Malignancy	9 (4%)
6.	Drug addiction	66 (31%)
7.	Suicidal tendency	10 (7%)
8.	Organ toxicity	11 (5%)
9.	Bleeding risk	4 (2%)
10.	Respiratory depression	8 (4%)
11.	Loss of bone mineral density	1 (0.4%)
12.	Food-drug interaction	1(0.4%)
13.	Increased mortality	4 (2%)
14.	Acid-base imbalance	8 (4%)
15.	Electrolyte abnormalities	7 (3%)
16.	Dehydration	1(0.4%)
17.	Complex sleep behaviors	3 (1%)
18.	Growth inhibition	1 (0.4%)
19.	Not approved for use in pediatric/elderly patients	2 (0.9%)
20.	Drug-drug interactions	1(0.4%)
21.	Drug overdose warnings	2 (0.9%)
22.	Radiation exposure with radioactive diagnostic agent use	1(0.4%)

Clinical significance of the black box warnings issued for commonly used drugs

Table 4 illustrates the clinical significance of the black box warnings for common drugs used in day-to-day practice to aid physicians in improved decision-making for patient safety [7]. Some of the examples of the drugs were misoprostol, a routinely used drug for obstetric purposes with a risk of fetal birth defects. Montelukast was issued with a serious warning of suicidal ideation.

**Table 4:** Clinical significance of the black box warnings for commonly used drugs Source: [7] The results were obtained from the cited reference.

S.No	Drugs	Class	Black box warning	Clinical significance
1.	Misoprostol	Prostaglandin analogs	Fetal birth defects in pregnant women, Risk of premature birth, Uterine rupture	Patients must be advised of the abortifacient properties and warned not to give the drugs to others. It should not be used to reduce the risk of NSAID-induced ulcers in pregnant women.
2.	Ethinyl estradiol; Levonorgestrel	Combined oral contraceptives (COC)	Hepatic enzyme elevation with hepatitis C drug combinations	It is contraindicated in females with acute viral hepatitis or severe (decompensated) cirrhosis of the liver. Acute liver test abnormalities may necessitate the discontinuation of the drug until the liver tests return to normal and causation has been excluded. Discontinue this drug if jaundice develops.
3.	Clopidogrel bisulfate	Antiplatelet drug	Diminished antiplatelet effect in patients with two loss-of-function alleles of the CYP2C19 gene (CYP2C19 poor metabolizers)	Tests are available to identify patients who are CYP2C19-poor metabolizers. Consider the use of another platelet P2Y12 inhibitor in patients identified as CYP2C19-poor metabolizers.
4.	Diclofenac sodium	Non-steroidal anti-inflammatory drugs (NSAIDs)	Risk of cardiovascular events and gastrointestinal events	Elderly patients and patients with a prior history of peptic ulcer disease and/or GI bleeding are at greater risk for serious GI events. Thus, it should be avoided in these patients.
5.	Montelukast sodium	Leukotriene receptor antagonist	Serious neuropsychiatric events-agitation, aggression, depression, sleep disturbances, suicidal thoughts, and behavior	Advise patients and/or caregivers to be alert for changes in behavior or new neuropsychiatric symptoms when taking montelukast sodium. If changes in behavior are observed, or if new symptoms or suicidal thoughts and/or behavior occur, advise patients to discontinue the drug and contact a healthcare provider immediately.
6.	Zolpidem	Sedative hypnotic	Complex sleep behaviors like sleep-walking, sleep-driving, etc.	Some of these events may result in serious injuries, including death. Discontinue the drug immediately if a patient experiences a complex sleep behavior.
7.	Levofloxacin, ciprofloxacin, gemifloxacin, moxifloxacin	Antimicrobial agent - Fluoroquinolone	Serious adverse reactions, including tendinitis, tendon rupture, peripheral neuropathy, central nervous system effects, and exacerbation of myasthenia gravis.	Discontinue fluoroquinolones immediately and avoid the use of fluoroquinolones, in patients who experience any of the mentioned serious adverse reactions. Avoid in patients with a known history of myasthenia gravis.
8.	Levothyroxine sodium	Anti-thyroid drug	Not for the treatment of weight loss.	In euthyroid patients, doses within the range of daily hormonal requirements are ineffective for weight reduction. Larger doses may produce serious or even life-threatening manifestations of toxicity, particularly when given in association with sympathomimetic amines such as those used for their anorectic effects.
9.	Tramadol hydrochloride	Opioid analgesic	Addiction, abuse, and misuse potential; risk of life-threatening respiratory depression.	Tramadol hydrochloride exposes patients and other users to the risks of opioid addiction, abuse, and misuse, which can lead to overdose and death. Assess each patient’s risk before prescribing, and monitor all patients regularly for the development of these behaviors and conditions.
10.	Alprazolam	Sedative hypnotic	Risks from concomitant use with opioids; abuse, misuse, and addiction; and dependence and withdrawal reactions	Reserve concomitant prescribing of these drugs for patients for whom alternative treatment options are inadequate. Limit dosages and durations to the minimum required. Follow patients for signs and symptoms of respiratory depression and sedation.
11.	Chlorpheniramine maleate; codeine phosphate	Antihistaminic/opioid antitussive	Ultra-rapid metabolism of codeine and other risk factors for life-threatening respiratory depression in children; risks from concomitant use with benzodiazepines or other central nervous system (CNS) depressants.	Contraindicated in all children younger than 12 years of age, as they are more susceptible to the respiratory depressant effects of codeine.
12.	Guaifenesin; hydrocodone bitartarate	Opioid expectorant	Addiction, abuse, and misuse potential; risk of life-threatening respiratory depression.	Contraindicated in all children younger than six years of age, significant respiratory depression, acute or severe bronchial asthma in an unmonitored setting or the absence of resuscitative equipment, and known or suspected gastrointestinal obstruction, including paralytic ileus.

Discussion

The black box warning is essential to highlight the serious adverse effects of a drug. It alerts health-care professionals to exercise caution before prescribing the drug [8]. On reviewing the black box warnings issued in the last 10 years, the greater number of warnings were issued in the year 2023 (40%) followed by 2022 (12%). Compared to newly added warnings (30%), the revision of the existing warnings (70%) was a larger number. The majority of the warnings were provided based on the post-marketing studies (78%) compared to pre-marketing studies (22%). The neuropsychiatric warning, addiction potential (31%), and hypersensitivity reactions (12%) were more in number compared to other warnings [9].

Upon reviewing the list of black box warnings on the official US FDA website, there were a total of 462 entries during the study period. However, there were multiple entries of the same drug with different brands but with the same black box warnings. For instance, the amphetamine derivatives, such as methamphetamine, dextroamphetamine, etc., were repeated with the same warning of addiction and abuse potential [10]. Similarly, different products of the radiocontrast media like gadoteridol, gadoterate meglumine, gadobutrol, etc., were given a warning of not to be given by intrathecal route for the risk of encephalopathy, seizures, coma, and death [11]. The oxycodone-containing combinations were issued a black box warning of addiction, abuse, and misuse potential, with life-threatening respiratory depression in case of overdose [12]. These could have been combined to avoid the misconception of multiple warnings. Instead, if the black box warning varies with different brands, it could have been highlighted in a separate section for better comprehension. The search term utilized to retrieve the information regarding black box warnings stated in the methodology was ‘Boxed warnings.’ Yet, the search results included the other sections such as warnings and precautions, use in specific populations, adverse reactions, etc., Thus, the section about the boxed warnings should have been restricted to only the black box warnings on the US FDA official website. This review compiled a wide range of black box warnings issued in the last decade at a single platform for the readers to contemplate.

In addition, this analysis included the black box warnings removed during the last 10 years. It includes the following examples, the boxed warning of potential risk of osteosarcoma for teriparatide was removed in 2020 [13]. The previously issued black box warning of angina pectoris on abrupt cessation of therapy for propranolol was removed in the year 2021 and shifted to the warnings and precautions section [14]. The black box warning of lower limb amputation was removed from the label of canagliflozin in 2020 after the FDA review of new data from the clinical trials suggesting that the risk of amputation is lower than previously described with appropriate monitoring [15].

The newly added warnings during the last decade included the risk of suicidal ideation due to montelukast. Regarding this concern, parents of children who suffered psychiatric disorders after taking montelukast are suing the pharmaceutical firm for allegedly designing a defective drug, negligence, and failure to adequately warn about mental health risks. Lawsuits claim that the marketing authorization holder should have known about these risks before marketing the drug in 1998. The FDA's addition of a black box warning in March 2020, highlighting serious neuropsychiatric side effects, prompted the lawsuits [16]. A study by Abdelkader S et al. compared the mental health adverse event reports in the pediatric population taking montelukast before and after the issuance of the black box warning for montelukast [17]. FDA reinforced montelukast warnings due to mental health risks, including suicides, despite limited new data. A boxed warning was introduced to underscore these concerns. Montelukast's use as a first-line treatment, especially for mild symptoms, is discouraged due to safer alternatives. Enhanced awareness among healthcare providers and patients is crucial to mitigate these risks effectively. This decision reflects a comprehensive evaluation of available evidence and prioritizes patient safety in medication management [18].

The commonly used sleep aids in current clinical practice like zolpidem and zaleplon were given a warning of complex sleep behaviors like sleepwalking and sleep driving, which may cause serious injuries [19]. A systematic literature review of several case reports and observational studies was conducted to investigate zolpidem's role in inducing complex sleep behaviors. On causality assessment, around 88% of cases were found to have a probable association of complex sleep behaviors with zolpidem. The review suggests that while zolpidem can be effective in treating insomnia, it also poses risks of inducing complex sleep behaviors, highlighting the need for cautious prescribing and monitoring of this medication [20].

The common class of drugs belongs to the antimicrobials (18%) followed by anticancer and immunosuppressant drugs (15%), opioid analgesics (8%), and sedative hypnotics (7%). In the antimicrobials, the majority were antiviral drugs for hepatitis like the combination containing sofosbuvir, elbasvir, etc., with the risk of reactivation of hepatitis in patients co-infected with hepatitis-B and hepatitis-C. HBV reactivation has been reported in HCV/HBV coinfected patients who were undergoing or had completed treatment with HCV direct-acting antivirals and were not receiving HBV antiviral therapy. Some cases have resulted in fulminant hepatitis, hepatic failure, and death. Therefore, monitoring is mandatory for HCV/HBV coinfected patients for hepatitis flare or HBV reactivation during HCV treatment and post-treatment follow-up [21].

Misoprostol, a medication commonly used in obstetrics to induce labor and terminate pregnancy medically, particularly for mid-trimester abortions, has a warning about birth abnormalities in the fetus. Therefore, misoprostol should not be used for any causes other than those listed above, such as treating NSAID-induced peptic ulcers in women who are of reproductive age. Since this warning is related to the vulnerable population, it should be spotlighted [22].

While guaifenesin-containing cough expectorants are commonly prescribed for pediatric patients in clinical settings, physicians must be mindful of the associated warning of the risk of fatal respiratory depression, particularly in children under the age of six. This awareness is essential to ensure the safe and appropriate use of such medications in pediatric populations. Parents or caregivers should be educated on administering cough syrup in therapeutic doses to children, emphasizing the importance of following prescribed guidelines rather than administering it deliberately [23].

Thus, this review provides condensed information on the black box warnings issued for the drugs in the last decade. It will help the health-care professionals to be diligent about the drugs and their warnings before prescribing. The clinicians should effectively communicate the black box warnings of the drugs to the patients for appropriate and safe use. It can encourage clinicians to explore a broader range of safer alternative medications rather than choosing those with black box warnings. Supposing there are no safer alternatives available and the patient’s condition warrants the use of drugs with black box warnings, the treating physicians should closely monitor the patients for any adverse events. As the concept of 'black box warning' gains attention, it will inspire healthcare professionals to stay current with their knowledge of medications for patient safety and adhere to the standard treatment guidelines.

Strengths and limitations

This review scrutinized the black box warnings issued over the last decade. It highlights the newly issued black box warnings over the last 10 years. Yet, this review comprised only the clinical significance of the commonly used drugs in clinical set-ups. Also, this review was restricted only to the US FDA's official website and not the other regulatory agencies such as the European Medical Association (EMA), Central Drugs Standard Control Organisation (CDSCO), etc.

## Conclusions

The black box warning serves as a vital tool for emphasizing the severe adverse effects of a drug. This review comprised a total of 225 black box warnings issued during the last decade. Neuropsychiatric warnings like addiction potential, suicidal tendencies, and hypersensitivity reactions accounted for the majority of the warnings. The antimicrobial agents received the maximum number of black box warnings during the study period. The commonly encountered drugs in clinical practice with black box warnings included the combination of guaifenesin; hydrocodone bitartrate, zolpidem, and montelukast. Having prior knowledge of black box warnings aids in mitigating serious adverse effects and providing better patient care.

## References

[1] (2024). Black box warnings. https://www.drugwatch.com/fda/black-box-warnings/#sources.

[2] (2024). Warnings and precautions, contraindications, and boxed warning sections of labeling for human prescription drug and biological products — content and format. https://www.fda.gov/regulatory-information/search-fda-guidance-documents/warnings-and-precautions-contraindications-and-boxed-warning-sections-labeling-human-prescription.

[3] Murphy S, Roberts R (2006). "Black box" 101: how the Food and Drug Administration evaluates, communicates, and manages drug benefit/risk. J Allergy Clin Immunol.

[4] (2024). CFR - Code of Federal Regulations Title 21. https://www.accessdata.fda.gov/scripts/cdrh/cfdocs/cfcfr/CFRSearch.cfm?CFRPart=201.

[5] Yeh JS, Sarpatwari A, Kesselheim AS (2016). Ethical and practical considerations in removing black box warnings from drug labels. Drug Saf.

[6] Frank C, Himmelstein DU, Woolhandler S (2014). Era of faster FDA drug approval has also seen increased black-box warnings and market withdrawals. Health Aff (Millwood).

[7] (2024). Drug safety-related labeling changes (SrLC). https://www.accessdata.fda.gov/scripts/cder/safetylabelingchanges/index.cfm?event=searchResult.page.

[8] Delong C, Preuss CV (2023). Box Warning. StatPearls.

[9] Lo CW, Pathadka S, Qin SX (2023). Neuropsychiatric events associated with montelukast in patients with asthma: a systematic review. Eur Respir Rev.

[10] (2024). FDA updating warnings to improve safe use of prescription stimulants used to treat ADHD and other conditions. https://www.fda.gov/drugs/drug-safety-and-availability/fda-updating-warnings-improve-safe-use-prescription-stimulants-used-treat-adhd-and-other-conditions.

[11] (2024). Drug label information. https://dailymed.nlm.nih.gov/dailymed/lookup.cfm?setid=cd718d1c-f813-4114-a50d-c744ff7eb1bf.

[12] (2024). FDA requires ‘black box’ warning on painkillers. https://edition.cnn.com/2016/03/22/health/fda-opioid-black-box-warning/index.html.

[13] Krege JH, Gilsenan AW, Komacko JL, Kellier-Steele N (2022). Teriparatide and osteosarcoma risk: history, science, elimination of boxed warning, and other label updates. JBMR Plus.

[14] Lasser KE, Allen PD, Woolhandler SJ, Himmelstein DU, Wolfe SM, Bor DH (2002). Timing of new black box warnings and withdrawals for prescription medications. JAMA.

[15] Aschenbrenner DS (2020). Boxed warning removed from canagliflozin labeling. Am J Nurs.

[16] (2024). Singulair black box warning. https://www.drugwatch.com/singulair/black-box-warning/.

[17] Abdelkader S, Hendrix-Dicken AD, Condren M (2023). The impact of Montelukast’s black box warning on pediatric mental health adverse event reports. J Pediatr Pharmacol Ther.

[18] 17] C. for D (2024). FDA requires boxed warning about serious mental health side effects for asthma and allergy drug montelukast (Singulair); advises restricting use for allergic rhinitis. https://www.fda.gov/drugs/drug-safety-and-availability/fda-requires-boxed-warning-about-serious-mental-health-side-effects-asthma-and-allergy-drug.

[19] 18] C. for D (2024). FDA adds boxed warning for risk of serious injuries caused by sleepwalking with certain prescription insomnia medicines. https://www.fda.gov/drugs/drug-safety-and-availability/fda-adds-boxed-warning-risk-serious-injuries-caused-sleepwalking-certain-prescription-insomnia.

[20] Mittal N, Mittal R, Gupta MC (2021). Zolpidem for insomnia: a double-edged sword. A systematic literature review on zolpidem-induced complex sleep behaviors. Indian J Psychol Med.

[21] Pockros PJ (2017). Black box warning for possible HBV reactivation during DAA therapy for chronic HCV infection. Gastroenterol Hepatol (N Y).

[22] (2024). CYTOTEC® (misoprostol) boxed warning. Pfizer Medical Information. https://labeling.pfizer.com/ShowLabeling.aspx?format=PDF&id=559.

[23] Halmo LS, Wang GS, Reynolds KM (2021). Pediatric fatalities associated with over-the-counter cough and cold medications. Pediatrics.

